# Second Annual Report to the General Assembly of Kentucky, Relating to the Registry and Returns of the Births, Marriages, and Deaths, from January 1, 1853, to December 31, 1853

**Published:** 1855-04

**Authors:** 


Art. VI.—*Second Annual Report to the General Assembly of Kentucky relating to the Registry and Returns of the Births, Marriages, and Deaths, from January* 1, 1853, *to December* 31, 1853.

---

To the unflagging industry of Dr. W. L. Sutton, whose zeal in the cause of vital statistics is so well known and so highly appreciated, the profession is indebted for a second report on the Births, Marriages, and Deaths in Kentucky; and although the compiler of these interesting details has to complain that the returns are not yet as complete as they should be, that some counties have failed to report the number of births and others the number of deaths, still he finds some evidence of improvement in the work of registration, and has been able to present a valuable summary of facts in this report. If physicians could only be awakened to the importance of the Registration Act, Kentucky might soon be on a footing with Massachusetts or England in respect to the amplitude of her vital statistics, and Dr. Sutton thinks he has indications of an increasing interest in the profession on the subject. With a population of a million, the returns of the Assessors show only nine thousand four hundred and eleven deaths in Kentucky during the year 1853. This shows great negligence, for it is impossible to believe that these numbers make any near approach to accuracy; Dr. Sutton supposes the numbers might be doubled, and still the state of health indicated by them would be satisfactory. The returns for 1853 agree with those of the former year in demonstrating that the average duration of life in Kentucky, as compared with the most favored countries as to health, is short, but then it is to be considered that the proportion of children under five years of age in this State is unusually large, and the mortality of this class is known to be great in all communities.

Of the deaths reported, the causes of 7,333 are assigned, and the summary under this head forms as interesting an extract as we could make from this meritorious report:—

"Upon referring to table X to learn the proportion of deaths from particular causes, we shall be forcibly struck with the great mortality from diseases of the first class—*Zymotics*. Although the proportion is considerably (8·41 per cent.) less than last year, it is still very large—no less than 48·01 per cent. Standing at the head of this class is *Cholera*, which by the first report gave 722 deaths, but which now presents but 49, among which are several cases of cholera morbus. *Cholera Infantum*, and *Diarrhœ* also return a small mortality. But *Croup*, though less in number, (390,) gives a greater per cent. than in the first report—having caused 5·18 per cent. of deaths from known causes. Of the 390 cases, 201 were males, 186 females.

"*Dysentery* presents itself as the greatest scourge of our state. Though only one-half as many are reported this year as last, yet it is considerably ahead of any other disease. This disease afflicts Massachusetts to a very great extent also. It caused an average of over 8 per cent. for 10 years, 1841–50; and from May 1, 1848, to December 31, 1850, it caused no less than 9,126 deaths. The whole number returned this year is 961, of which 486 were males, and 474 females, showing that females were the greatest sufferers from this disease. There were 747 whites to 214 colored—making a slight excess against the whites.

"Under the head of *Intermittent Fever* are included all cases of congestive fever and congestive chills, because those forms of of disease are usually intermittent in character. The few cases of yellow fever are classed with remittent. Of 'fever' and 'continued fever' together, there were 924 cases, being about the same per cent. as last year, (12·74). There were 476 males, and 456 females; again a balance against the females. There were 719 whites, and 214 colored, very nearly in equal proportions.

"*Scarlet Fever* was one of the few diseases which proved more fatal in 1853 than in 1852. The number was 306, or 4·17 per cent. Both *Hooping-cough* and *Measles* fell off. The first to 180; the other to 284. *Milk-sickness* again presented a smaller mortality than was expected—only 9 in all. No other disease in this class was very fatal.

"Class II, made up of a considerable number of diseases, presents only 505 in all, of which *Dropsy* caused 216. Of these 146

were whites, and 70 colored—more fatal on the colored; 113 were males, and 103 females. Next in mortality is *Scrofula*, which caused 115 deaths; of which 39 were whites, and 67 colored—greatly more fatal to the colored; 57 males, and 59 females—more fatal to females.

"Class III. *Diseases of the Nervous System*, present a mortality something greater than the last—having caused 557 deaths. The most fatal disease in the list was *Inflammation of the Brain*, to which 218 deaths, or 2·99 per cent. of deaths, are ascribed. Of these, 167 were whites, 51 colored; 109 males, 106 females; being a pretty fair division between white and colored; but contrary to most diseases of early life, giving a preponderance against the females. Next is *Convulsions* which caused 98 deaths—59 whites and 39 colored; an excess of colored; and 54 males and 44 females; a considerable excess of males.

"Class IV. *Diseases of the Respiratory System*, usually are next to the Zymotics in mortality. We find 1,586 deaths, or 21·63 per cent. ascribed to this class; considerably more than the proportion of last year, notwithstanding the absolute number of deaths is 209 less. *Consumption* caused 846 deaths, being something more than all other diseases of this class. Of these 633 were whites, and 213 colored—a small excess against the colored; 304 males, and 542 females; a very great preponderance against the females.

"This is, in various respects, a very important disease. It selects its victims at all ages, seeming to have no preference except for the fairest, most interesting and promising; no rank or age is exempt; and it carries its victims to the grave alike during every month of the year. It, in many countries, causes more deaths than any other single disease. It is one which is exceedingly rarely cured. It is to be prevented, not cured. And it is one which, to a great extent, can be avoided. We have been accustomed to believe that England is particularly obnoxious to consumption—to hear and believe that 50,000 persons die annually of consumption in England. And truly, this sounds largely. But we forget that the population of England is very great, and that these 50,000 deaths are only 13 per cent. of her deaths. In Massachusetts, Connecticut, and New York, consumption causes over 20 per cent. of deaths. In our own state in the first report, it caused a mortality of 957, or 9·20 per cent. of deaths from all known causes; and in the present, 846 or 11·45 per cent. It appears, therefore, from the two, that Kentucky is favorably situated to guard against this disease. But no country or climate will do as much to prevent it as a proper attention to the laws of health.

"*Pneumonia* is next to consumption in fatality, having caused 383 deaths, or 5·22 per cent.; 272 whites, and 111 colored; 221 males and 161 females, a large proportion of whites, and a large proportion of males. *Catarrh*, under which are included 'colds,' is the only other disease of this class which is deemed necessary to notice. It caused 130 deaths, or 1·77 per cent.

"CLASS V. *Diseases of the Circulatory System*, cause but 50 deaths, which were divided among a considerable number of affections of those organs.

"CLASS VI. *Diseases of the Digestive System*, altogether occasioned 329 deaths, or 4·47 per cent. As a class, the mortality is small compared with other countries; nor does any one disease cut much figure. *Enteritis* caused 98 deaths, and *Worms* 73.

"CLASS VII. *Diseases of the Urinary System*, caused but 33 deaths in all; of which 19 are attributed to *Gravel*.

"CLASS VIII. *Diseases of the Generative System*, caused 129 deaths, or 1·76 per cent. being one of the few classes which presents as great a number of deaths as last year's report. As usual, the principal mortality was from *Child-birth* and *Puerperal Fever*. The former caused 66 deaths, one-third more than last year. Of these 55 were whites and 11 colored; quite an undue proportion of whites. Among the whites one death from childbirth occurred to 358 births; among the colored one in 473, reversing the proportions of last year, and one in 379 in the whole number of births. From *Puerperal Fever* there were 43 deaths, of which 31 were whites and 12 colored; being one to 638·58 white births, and one in 434 colored. The two causes jointly caused one death in 229 births.

"In the first report it was said: 'Diseases of the generative system give a rather large proportion,' childbirth and puerperal fever constituting nearly the whole amount. But in comparing deaths in Kentucky from the parturient state, with those in other countries, the result will be found not so unfavorable as was at first supposed. By referring to the Tenth Massachusetts Report it appears that for three years the average was 200 deaths annually from these two causes. But the English report is greatly worse than either. In the third annual report (English,) it is said, 'childbirth was fatal to 2,915 women. Out of 1,000,000 women living, 368 died from this cause in 1838, and 372 in 1839. About five births in 1,000 are fatal to the mother.' Again, (Fourth Annual Report,): 'The deaths in child-bed were 2,811, 2,915, and 2,989 in three years. * * To about 187 children born alive, *one mother died.*' Again, (Fifth Report,): 'Of childbirth, 3,195 died in the town, and 1,806 in the country districts; the excess in towns was more than a thousand lives; the mortality was 231 and 137,' (to the 1,000,000 living). This must have embraced puerperal fever and all the ills of the parturient state; and truly it is an appalling list.

"Class IX. *Diseases of the Locomotive System*, give a mortality of 61, something greater than last year.

"Class X. *Diseases of the Integumentary System*, present a mortality of 23, 17 of which were *Ulcers*.

"Class XI. *Old Age*, gives only 198; being 46 less than last year.

"Class XII. *From External Causes*, furnish 341 deaths; being 96 less than last year; 73 are ascribed to *Accidents*, of which 57 were whites, and 16 colored; 51 males and 22 females. *Burns* are charged with 46 deaths, of which 26 were whites and 20 colored, quite an excess of colored; 20 were males, and 26 females. Thirty-three were *Drowned;* of which 27, (24 males, and 3 females,) were white, 6 black males. *Intemperance* is charged with 19 deaths; 16 white males, and 3 black males. Twenty-four are reported as killed *Accidentally;* 16 whites, 15 males and 1 female, 8 colored males. Seventeen were killed by *Design;* 12 whites, 11 males, and one female, 5 colored males. This is a considerable abatement in both the accidentally and designedly killed—being as 43 to 79.

"*Suffocation* is charged with 81 deaths, of these 15 were whites, 11 males and 4 females, 66 colored, 34 males, and 32 females. Of these 81, 78 were under 5 years.

"This twelfth class is a pretty good barometer of the carefulness or negligence of a community. Many of the cases might evidently have been prevented by proper care. It bears heavily upon the improvident class—the colored. Of this, suffocation is a strong example. Thus, 146 are returned as dying of this cause in the two years of reports; of these 114 were colored, and only 32 whites."

